# Supplementary material for: Predictive Value of SLCO1B1 c.521T>C Polymorphism on Observed Changes in the Treatment of 1136 Statin-Users
Source: Genes (Basel). 2023 Feb 10;14(2):456. doi: 10.3390/genes14020456 (PMC9957000; doi:10.3390/genes14020456)
Supplement: Supplementary file 1 [file genes-14-00456-s001.zip › genes-2192843-supplementary.pdf]

Predictive value of *SLCO1B1* c.521T>C polymorphism on observed changes in the treatment of 1136 statin-users

Supplementary

Supplementary Table S1. Sample size calculation based on Link *et al.* (2008) in a population of patients receiving 80 mg of simvastatin daily.

	Controls	Cases	Total
T	157	93	250
C	23	77	100
Total	180	170	350
C allele proportion	13%	45%	
Needed sample size	39	41	80

Supplementary Table S2. Calculation Hardy Weinberg Equilibrium

TT	789
TC	316
CC	31

TTexp	789,4445
TCexp	315,1109
CCexp	31,44454

p	0,833627	n	1136
q	0,166373		

Chi-squared	0,009	p-value	0,995488
